# Research Review: The effect of school‐based suicide prevention on suicidal ideation and suicide attempts and the role of intervention and contextual factors among adolescents: a meta‐analysis and meta‐regression

**DOI:** 10.1111/jcpp.13598

**Published:** 2022-03-15

**Authors:** Eibhlin H. Walsh, Jennifer McMahon, Matthew P. Herring

**Affiliations:** ^1^ School, Child & Youth (SCY) Mental Health and Wellbeing Research Lab National Institute of Studies in Education Health Research Institute University of Limerick Limerick Ireland; ^2^ 8808 Department of Psychology University of Limerick Limerick Ireland; ^3^ Physical Activity for Health Cluster, Health Research Institute University of Limerick Limerick Ireland; ^4^ 8808 Department of Physical Education and Sports Sciences University of Limerick Limerick Ireland

**Keywords:** adolescence, meta‐analysis, post‐primary school‐based suicide prevention, Suicidal thoughts and behaviours

## Abstract

**Background:**

Globally, suicide is the fourth highest cause of adolescent mortality (*Suicide*: https://www.who.int/news‐room/fact‐sheets/detail/suicide). The effects of post‐primary school‐based suicide prevention (PSSP) on adolescent suicidal thoughts and behaviours (STBs) have not been comprehensively synthesised. We aim to estimate the population effect for PSSP interventions on adolescent STBs and explore how intervention effects vary based on intervention and contextual moderators.

**Methods:**

Searches of PsycINFO, Medline, Education Source, ERIC, Web of Science, and the Cochrane Central Register of Controlled Trials identified cluster randomised trials examining the effectiveness of PSSP on adolescent STBs. The Cochrane Risk of Bias tool assessed bias. Crude and adjusted back‐transformed odds ratios (ORs) were calculated. Multilevel random‐effects models accounted for dependencies of effects. Univariate meta‐regression explored variability of intervention and contextual moderators on pooled effects.

**Results:**

There were 19 and 12 effects for suicidal ideation (SI) and suicide attempts (SA). Compared with controls, interventions were associated with 13% (OR = 0.87, 95%CI [0.78, 0.96]) and 34% (OR = 0.66, 95%CI [0.47, 0.91]) lower crude odds reductions for SI and SA, respectively. Effects were similar for adjusted SI (OR = 0.85, 95%CI [0.75, 0.95]) and SA (OR = 0.72, 95%CI [0.59, 0.87]) models. Within‐study (0.20–9.10%) and between‐study (0–51.20%) heterogeneity ranged for crude and adjusted SA models and SI heterogeneity was 0%. Moderator analyses did not vary SA effects (*p*s > .05).

**Conclusions:**

This meta‐analysis contributes to the PSSP evidence‐base by demonstrating that PSSP targeting STBs as both primary intervention outcomes and with other health and well‐being outcomes reduced SI and SA among 33,155 adolescents attending 329 schools, compared to controls. The number needed to treat estimates suggests the potential of reducing the incidence of SA and SI in one adolescent by implementing PSSP in 1–2 classrooms, supporting PSSP as a clinically relevant suicide prevention strategy. Although moderator analyses were nonsignificant and contained a small number of trials, larger SA effect sizes support particular effectiveness for interventions of a duration of ≤1 week, involving multiple stakeholders and with a 12‐month follow‐up.

## Introduction

Globally, suicide is the fourth highest cause of mortality in 15–19‐year‐olds (World Health Organisation [WHO], 2021). From 2008 to 2018 deaths by suicide in the United States have continued to rise in 15–24‐ and 10–14‐year‐olds by 42% and 130%, respectively (Centers for Disease Control & Prevention [CDC], 2020). Suicidal thoughts and behaviours (STBs) include suicidal ideation (SI) (i.e., thoughts of killing oneself) and suicide attempts (SA) (i.e., acts of intention to end one’s life not resulting in death) (Goldsmith, 2002). Twelve‐month prevalence rates of adolescent SI and SA are estimated at 16.5% and 16.4%, respectively (Tang et al., 2020). Thus, identifying interventions effective in reducing adolescent STBs is needed.

Schools are logical contexts for adolescent suicide prevention, given their wide‐ranging capacity for reaching adolescents and mandate enactment (White, Morris, & Hinbest, 2012). Post‐primary school‐based suicide prevention (PSSP) strategies traditionally have been categorised as universal (e.g., awareness programmes), selective (e.g., screening), and indicated (e.g., interventions for high‐risk adolescents) (Goldsmith, 2002). We define PSSP as interventions located in post‐primary school‐based settings, which target STBs as both primary intervention outcomes and with other health and well‐being outcomes, consistent with recommendations for: (1) targeting problems associated with suicide to reduce suicide (Large, Ryan, Carter, & Kapur, 2017), (2) prevention embedded in general mental health strategies (Miller, Eckert, & Mazza, 2009), and (3) upstream PSSP interventions which reduce STBs indirectly by addressing risk and protective processes (Wyman, 2014). Previous PSSP reviews selected interventions designed to target youth STBs (Miller et al., 2009), and excluded general mental health programmes (Surgenor, Quinn, & Hughes, 2016) and interventions that did not primarily target STBs (Robinson et al., 2018). A recent meta‐analysis extended inclusion criteria to include interventions in primary and post‐primary school settings that focus on risk factors of STBs to target STBs in children and adolescents, and preliminarily supported these interventions to have preventative potential (Gijzen, Rasing, Creemers, Engels, & Smit, 2022). As such, expanding the focus of PSSP interventions is crucial to clarifying the effectiveness of PSSP for adolescent STBs.

The global policy recommends the implementation of evidence‐based PSSP (WHO, 2018). Rigorous systematic reviews and meta‐analyses of randomised studies are pivotal in informing evidence‐based insights (Mittman, 2004), with these studies amongst the highest level of evidence informing intervention (Burns, Rohrich, & Chung, 2011). Meta‐analytic evidence of PSSP effectiveness for reducing adolescent STBs is currently limited: syntheses of studies employing cluster randomised trial (CRT) designs, demonstrating school‐based suicide prevention effectiveness on SI included samples engaging in interventions as children (Pistone, Beckman, Eriksson, Lagerlof, & Sager, 2019) and containing young adults (Pistone et al., 2019; Robinson et al., 2018). Recent meta‐analyses including PSSP interventions demonstrate small composite effects of reduced STBs postintervention based on randomised (Gijzen et al., 2022) and non‐randomised (Brann, Baker, Smith‐Millman, Watt, & DiOrio, 2021) studies. Rigorous composite effects of PSSP effectiveness on adolescent STBs based on CRTs are needed, consistent with recommendations that randomised and non‐randomised studies are generally synthesised separately, particularly when additional sources of heterogeneity associated with CRT designs would likely interact with the intervention, which is the case when schools and classrooms are randomisation units in PSSP research (Borenstein, Hedges, Higgins, & Rothstein, 2011; Donner & Klar, 2002).

Finally, reviews highlight the need to address gaps in understanding how intervention, school‐, and cultural‐level contextual factors may influence PSSP effectiveness (Hofstra et al., 2020; Miller et al., 2009; Robinson et al., 2018). Variation of PSSP effectiveness across settings supports the need for a greater understanding of how contextual and intervention factors vary PSSP effectiveness, to better understand how, where, and when PSSP strategies are most effective (Breet, Matooane, Tomlinson, & Bantjes, 2021).

### Objectives

This meta‐analysis addressed the need for rigorous estimation of the population effect of PSSP interventions targeting STBs as both primary intervention outcomes and with other health and well‐being outcomes on STBs in adolescents, evaluated using CRT designs. To address the gap in understanding how intervention and contextual factors vary PSSP effectiveness, we examined intervention type, intervention duration, follow‐up period, and stakeholder involvement in interventions as moderators, consistent with theoretically and empirically informed intervention and contextual factors in school‐based prevention research (Domitrovich et al., 2008).

## Methods

This review was preregistered with PROSPERO (ID: CRD42020168883). This study adhered to the PRISMA 2020 statement guidelines (see Table S1) (Page et al., 2021).

### Search strategy

In February 2020 and again in January 2021, PsycINFO, Medline, Education Source, ERIC, Web of Science, and the Cochrane Central Register of Controlled Trials were searched to identify studies published from database inception until January 2021, using combinations of keywords representing the following concepts: adolescent, postprimary school, intervention, and STBs (see Table S2). Consistent with the Population, Intervention, Control, Outcome and study design (PICOs) framework (Methley, Campbell, Chew‐Graham, McNally, & Cheraghi‐Sohi, 2014), studies were eligible if they: (a) included adolescents aged 11–19 years attending post‐primary school (‘Population’), (b) evaluated interventions conducted in post‐primary school settings, measuring STBs including SI, SA, and planning, and death by suicide (‘Intervention’ and ‘Outcomes’), (3) contained comparators including no intervention, other intervention, and wait‐list control (‘Control’), and (4) employed CRT study designs (‘study design’). Studies unavailable in English, non peer‐reviewed publications, and non‐CRT studies were excluded. Reference lists of screened studies were searched.

EW assessed study title and abstract eligibility. Eligible studies were fully screened in duplicate by EW and JMM using Rayyan (Ouzzani, Hammady, Fedorowicz, & Elmagarmid, 2016). Consensus was reached on disagreements. Cohen’s Kappa coefficient (Cohen, 1960) showed adequate agreement on original full‐text screening decisions by JMM and EW (*κ* = .72, *p* < .001). EW contacted 20 authors for unavailable study details and data (9 incomplete requests). Trials were excluded due to the evaluation of non school‐based interventions (Poppelaars et al., 2016) and unavailability of crude data (O'Leary‐Barrett et al., 2013; Orbach & Hanna, 1993; Randell, Eggert, & Pike, 2001) and for the first follow‐up (Vieland, Whittle, Garland, Hicks, & Shaffer, 1991).

### Data extraction and evaluation

Data were organised by PICOs and intervention and contextual factors. EW completed data extraction. EW and CB extracted intervention and contextual factors in duplicate. Available follow‐up outcome data and eligible interventions were extracted for synthesis inclusion. Cochrane Collaboration Risk of Bias Tool for CRTs assessed the risk of bias (Eldridge et al., 2016). The question ‘Were participants aware that they were in a trial?’ and ‘Bias in the measurement of the outcome’ domain were omitted for unsuitability to PSSP research. Four studies randomly assessed in duplicate by EW and MH demonstrated adequate agreement (*κ* = .63, *p* = .03).

#### Effects synthesis and analysis

Data were analysed with R statistical packages *metafor* (Viechtbauer, 2010) and *meta* (Schwarzer, 2007). Suicide planning (Schilling, Aseltine, & James, 2016; Schilling, Lawless, Buchanan, & Aseltine, 2014) and deaths by suicide (Wasserman et al., 2015) were not retained for meta‐analysis due to insufficient numbers of effects. Only trials which measured SA and SI were synthesised.

Crude log odds ratios (ORs) and sampling variances were calculated using trial proportions of SA and SI events (Viechtbauer, 2010). Where trial proportions were unavailable, crude ORs and corresponding *p*‐values (Shinde et al., 2020) and raw study data (Perry et al., 2014; Poppelaars et al., 2016) were used. Table S3 details missing data procedures. Corresponding trial effects adjusted for study characteristics (see Table S4) were pooled. Reported log ORs (Fekkes et al., 2016; Gould et al., 2005; Wasserman et al., 2015) and ORs (Shinde et al., 2020) and corresponding *p*‐values were extracted or calculated (Aseltine, James, Schilling, & Glanovsky, 2007; Schilling et al., 2014, 2016; Wyman et al., 2010), according to standard procedures (Altman & Bland, 2011; Restore, 2011). Where no adjustments were reported (Poppelaars et al., 2016; Vieland et al., 1991) or data were unavailable for SI (Perry et al., 2014, 2017) and SA (Schilling et al., 2014), crude log ORs were imputed.

#### Meta‐analysis and moderator analyses

Multilevel random effects univariate meta‐analytic and meta‐regression models estimated sampling error and population variance, with weighted least squares estimation and restricted estimation likelihood (Borenstein et al., 2011; Viechtbauer, 2010). To synthesise all available trials and follow‐ups, multilevel models incorporated a random effect for trials from the same studies containing dependent control and/or intervention participants (Perry et al., 2014, 2017; Poppelaars et al., 2016; Shinde et al., 2020; Wasserman et al., 2015), to account for the dependency of these effects (Fernandez‐Castilla et al., 2020; Viechtbauer, 2010).

Estimates of average pooled effects were obtained by fitting models to log ORs and back transformation to ORs through exponentiation (Viechtbauer, 2010), representing the likelihood of SI and SA in the pooled intervention group, compared to the pooled control group. Cochrane’s *Q* statistic and measures of consistency *I* ^2^ quantified between‐ and within‐study heterogeneity; *I*² < 40% and 30%–60% indicates low and moderate heterogeneity, respectively (Schünemann, Brożek, Guyatt, & Oxman, 2013). Forest plots weighted by their inverse variance depict the distribution of trial ORs. Numbers needed to treat (NNT) were calculated using patient expected event rate (PEER) baseline risks (Mendes, Alves, & Batel‐Marques, 2017), based on the 12‐month prevalence of SI (16.5%) and SA (16.4%) across 83 countries (Tang et al., 2020).

Meta‐regressions were not conducted where no heterogeneity was observed (Higgins & Thompson, 2002). Moderator reference categories were the lowest or negative category level. *A priori* moderators region and youth involvement contained *k* < 1 and were not examined as moderators (Schmidt, 2017). No school characteristics were examined as moderators as 11/12 studies sampled public schools, and few studies provided clear reporting of (a) school setting (Aseltine et al., 2007; Vieland et al., 1991; Wyman et al., 2010), (b) the gender profile of schools (Gould et al., 2005; Shinde et al., 2020), and (c) exact school sizes (Shinde et al., 2020; Vieland et al., 1991). When duration was presented as a range (Perry et al., 2014, 2017) the median was calculated. Stakeholder involvement included school personnel involved in delivering interventions. Meta‐regression models without the intercept term assessed the average OR for moderator levels and models with the intercept produced log OR contrasts (Viechtbauer, 2010). Publication bias of crude pooled log OR models was assessed by extending Egger’s Regression Test (Egger, Smith, Schneider, & Minder, 1997) to meta‐regression models, with sampling variances as the moderator (Viechtbauer, 2010). Crude SA and SI trial 95% confidence intervals (CIs), Cook’s Distance (Cook & Weisberg, 1982), and internally standardised residuals were examined to detect outliers. If outliers considerably impacted meta‐analysis conclusions exclusion was considered (Viechtbauer & Cheung, 2010).

## Results

**Figure 1:**
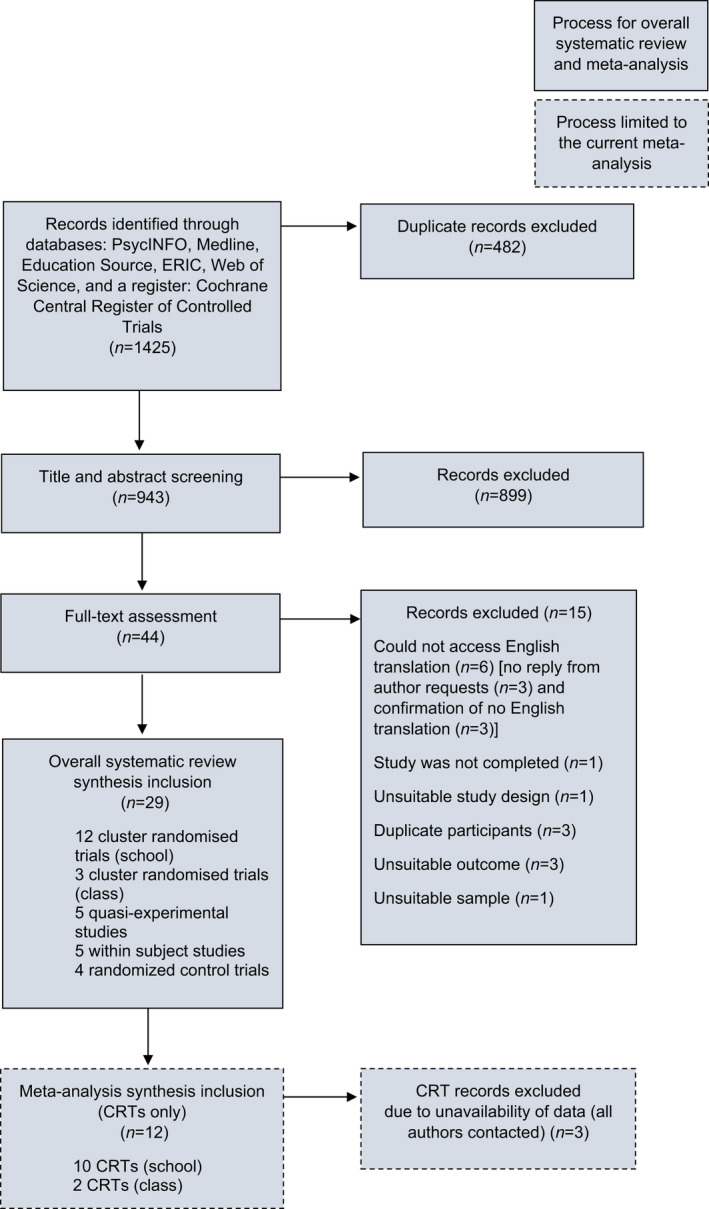
PRISMA 2020 flow diagram

Twelve studies were retained (see Figure 1). The lower‐bound 95%CIs of the teacher‐led trial by Shinde et al. (2020) were higher than the SA pooled effect upper‐bound 95%CI, indicating that this trial is not part of the ‘population’ effect (Harrer, Cuijpers, Furukawa, & Ebert, 2019). The trial’s Cook’s Distance (.07) and internally standardised residual (1.04) were the largest among all trials. The teacher‐led trial by Shinde and colleagues was excluded, as the comparison of coefficients for analyses including this trial (see Table S5) with the reported analyses indicated that this trial unusually influenced results.

There were 19 and 12 retained effects for SI and SA, respectively. Studies were published from 1991 to 2020, containing 33,155 participants aged 11–18 years, sampled across 329 schools. Some studies did not report age but reported school grade (Aseltine et al., 2007; Schilling et al., 2016) or did not report either (Schilling et al., 2014; Wyman et al., 2010). The largest (Wasserman et al., 2015) and smallest (Poppelaars et al., 2016) studies contained 11,110 and 208 participants, respectively. The median percentage of male participants across studies was 49%, calculated from 11/12 studies reporting gender percentages before and after participant dropout. Studies contained mixed‐gender samples, other than one female‐only sample (Poppelaars et al., 2016). Half of the studies were conducted in North America, and also in Europe, Australia, and Asia. The risk of bias assessments is presented in Table S3.

Interventions are fully described in Table S3 and trials were categorised as (a) universal (11/22 trials, including Headstrong, SPARX‐R, Strengthening Evidence base on scHool‐based intErventions for pRomoting adolescent health [SEHER], and Youth Aware of Mental Health [YAM]), (b) selective (3/22 trials, including Profscreen), (c) selective/indicated (4/22 trials, including Question, Persuade and Refer [QPR], Op Volle Kracht [OVK], and OVK/SPARX), and (d) universal/selective (4/22 trials including Signs of Suicide [SOS] and Sources of Strength). Most interventions (7/13) targeted SI and SA along with other health and well‐being outcomes (Fekkes et al., 2016; Perry et al., 2014, 2017; Poppelaars et al., 2016; Shinde et al., 2020; Wyman et al., 2010).

### Meta‐analysis

There were 22,195 and 20,984 dependent cases in the pooled control and interventions groups, respectively, for SA (control = 10,585 and intervention = 14,406) and 19,058 and 18,993 dependent cases in the pooled control and interventions groups, respectively, for SI (control = 7,086 and intervention = 12,043). Forest plots for SI and SA are presented in Figures S1 and S2, respectively (see Figures S3 and S4 for forest plots of adjusted counterparts for SI and SA, respectively). SI and SA were lower for pooled intervention groups in 13/19 and 9/12 of trials, respectively.

#### Suicidal ideation

For crude ORs, compared to the pooled control group, the pooled intervention group was associated with 13% lower odds of SI (OR = 0.87 [95%CI: 0.78, 0.96]). The composite effect was not heterogeneous (*Q*_18_ = 15.41, *p* = .63). Within‐study and between‐study heterogeneity = 0.0%. For adjusted ORs, OR = 0.85 [95%CI: 0.75, 0.95]; *Q*_18_ = 10.04, *p* = .93, and within‐study and between‐study heterogeneity = 0.0%.

#### Suicide attempts

For crude ORs, the pooled intervention group was associated with 34% lower odds of SA (OR = 0.66 [95%CI: 0.47, 0.91]. The composite effect was not heterogeneous (*Q*_10_ = 16.31, *p* = .13). Within‐study = 0.20% and between‐study heterogeneity = 51.20%. For adjusted ORs, OR = 0.72 [95%CI: 0.59, 0.87], *Q*_18_ = 10.46, *p* = .49) and within‐study = 9.10% and between‐study heterogeneity = 0%.

#### Number needed to treat

The crude PEER‐adjusted NNT was 55 (adjusted = 47) for SI and 20 (adjusted = 25) for SA.

#### Extended Egger’s regression test

Sampling variance as moderators were nonsignificant for crude SI (log OR = −0.14, *p* = .74 [95% CI: −0.98, 0.69]; QM_1_ = 0.11, *p* = .74; Q_17_ = 15.30, *p* = .57) and SA (log OR = 0.39, *p* = .68 [95%CI: −1.45, 2.24]; QM_1_ = 0.18, *p* = .68; Q_10_ = 16.30, *p* = .09), and adjusted SI (log OR = 0.14, *p* = .72 [95%CI: −0.64, 0.93]; QM_1_ = 0.13, *p* = .72; QE_17_ = 9.91, *p* = .91) and SA (log OR = 0.01, *p* = .94 [95%CI: −0.41, 0.44]; QM_1_ = 0.005, *p* = .94; QE_10_ = 10.46, *p* = .40).

### Meta‐regressions

Estimations of average SA odd reductions for trials at moderator categories are presented in Tables S6 and S7. Univariate meta‐regression for SA were nonsignificant for crude and adjusted models (all *p*s > .05).

## Discussion

This study addresses an important gap in estimating the population effect of PSSP interventions on SA and SI in adolescents using crude and adjusted estimates adjusted for putative confounders and exploring how intervention and contextual factors potentially vary PSSP intervention effectiveness. Compared to controls, PSSP interventions were associated with 13–15% and 28–34% lower odds of SI and SA, respectively, among 33,155 adolescents attending 329 schools. Intervention effects for SA did not vary based on *a priori* moderators, and homogeneity in SI models prohibited meta‐regression analyses.

### Intervention effectiveness

This meta‐analysis extends previous findings showing significant reductions in adolescent SI following engagement with PSSP interventions by an addition of 5 trials (Gijzen et al., 2022). Considering that lifetime prevalence rates predict that approximately one third of adolescents who experienced SI went on to develop a suicide plan and attempt suicide (Nock et al., 2013), identifying interventions effective in reducing adolescent SI, such as PSSP, is crucial. Present findings extend previous meta‐analysis findings demonstrating reductions in SA among adolescents following school‐based suicide prevention at 3‐month follow‐up (Pistone et al., 2019) and adolescents and young adults at postintervention and follow‐up (Robinson et al., 2018), by an additional nine trials.

Present findings extend the evidence‐base by synthesising a range of PSSP interventions targeting adolescent SA and SI as both primary intervention outcomes and with other health and well‐being outcomes, including universal, selective, and indicated PSSP, as well as interventions based on social and emotional learning, depression prevention, health promotion, and health and physical education curriculum. Because schools are likely to have limited resources to fund preventative interventions (Balaguru, Sharma, & Waheed, 2012), expanding the focus on PSSP is important for PSSP research. The composite effects reported here are critical to understanding the ‘true’ extent of PSSP effectiveness, as large sample sizes are needed to detect the effectiveness of PSSP interventions, given that base rates and incidences of STBs are low relative to the population (Goldsmith, 2002; Kapur & Gask, 2009). This is reflected in the discrepancy between nonsignificant trial effects and the significant composite effects for SA and SI herein.

Based on the PEER baseline risks, NNT for crude and adjusted models estimate that one less adolescent would have a SA and experience SI for every 20–25 and 47–55 adolescents engaging in PSSP, respectively. This suggests that implementing PSSP in two typically sized classrooms could prevent at least one SA and one incidence of SI. The clinical significance of the NNT for SA is comparable with the ED‐SAFE intervention for reducing SA in individuals in emergency department settings (NNT = 22) (Miller et al., 2017). Given the challenges of effectively tackling adolescent STBs with therapeutic interventions (Ougrin, Tranah, Stahl, Moran, & Rosenbaum Asarnow, 2015), cognitive‐behavioural interventions (Tarrier, Taylor, & Gooding, 2008) and multinational suicide prevention interventions (Matsubayashi & Ueda, 2011), the present findings have valuable clinical relevance for targeting adolescent STBs.

The next steps in the field should focus on how best to maximise dissemination of PSSP, including scaling, adoption, implementation, and sustainability (Flay et al., 2005). Considering that early adolescence is associated with STBs (van Vuuren, van der Wal, Cuijpers, & Chinapaw, 2020) and risk factors, including the onset of mental health difficulties, which further predict early school drop‐out (Jozefowicz‐Simbeni, 2008), earlier implementation of PSSP would increase the likelihood that adolescents who are most vulnerable receive PSSP.

### Meta‐regressions

Meta‐regressions did not support any significant moderators of the composite effects for crude and adjusted SA estimates. Notably larger SA odds reductions across crude and adjusted models were found for trial effects with 12‐month follow‐ups (*k* = 3, *n* = 1), in comparison to trials with ≤3 months and 17–20‐month follow‐ups. The 12‐month follow‐up effects were derived from universal, selective, and indicated PSSP interventions evaluated as part of the SEYLE study (Wasserman et al., 2015). Notwithstanding that these trials contain dependent control groups, future PSSP research should include a minimum of 12‐month follow‐ups for SA, as sufficient time may be needed to show the minimum detectable preventive potential of PSSP.

Additionally, larger SA crude and adjusted odd reductions were observed for studies containing interventions of a duration of ≤1 week (*k* = 4, *n* = 4), with 3/4 of these interventions examining the SOS programme, which infers programmatic effects. This suggests that interventions, may not have to be time‐intensive to be effective, which is practically important given scarce resources for school‐based health interventions (Ahern et al., 2018). Larger crude and adjusted odds reductions at the category of multistakeholder involvement (*k* = 4, *n* = 3) suggest that PSSP effectiveness could be enhanced through involving a range of school stakeholders in the delivery of PSSP interventions, including counsellors, teachers, and other school personnel, consistent with evidence that school personnel play critical roles in school‐based preventative strategies (Jozefowicz‐Simbeni, 2008; Page, Saumweber, Hall, Crookston, & West, 2013). Inconsistency of crude and adjusted effects for interventions that did not primarily target suicide, and the small number of effects at this moderator level (*k* = 2,*n* = 2) preclude the interpretation of the effectiveness of these interventions in comparison to interventions primarily targeting STBs (*k* = 10, *n* = 5) on SA, despite interventions which primarily targeted suicide demonstrating greater odd reductions in SA, suggesting greater effectiveness of these interventions.

### Risk of bias

Domains of bias assessed include randomisation process, participant and reporting selection, and fidelity. The Cochrane Collaboration Risk of Bias tools are amongst the most comprehensive assessment approaches of potential bias in randomised trials (Higgins et al., 2011). Half (6/12) of the included studies were determined as low risk of bias, with four studies rated as high risk of bias. Considering that study quality issues in reviews synthesising PSSP randomised and non‐randomised studies have been previously described (Pistone et al., 2019; Robinson et al., 2018), it is recommended that CRT studies are prioritised for determining the PSSP evidence‐base and are the standard design included in prospective meta‐analyses. Omission of nonrelevant questions may limit the validity of the risk of bias assessment but is similar to previous PSSP reviews (Robinson et al., 2018), suggesting the need for bias assessment tools with greater relevance to PSSP research.

Publication bias was not an issue, supported by nonsignificant moderator analyses of the adapted Egger’s Regression Test and the consistency of the magnitude of crude SA and SI effects, with effects adjusted for characteristics including demographics, baseline STBs, and mental health treatment. Selection bias was limited through synthesising all available follow‐up and relevant intervention effects in retained studies, and extends previous meta‐analyses synthesising PSSP effects, which have retained only the intervention follow‐up at postintervention (Brann et al., 2021) and longest‐term (Hofstra et al., 2020; Robinson et al., 2018) or conducted separate meta‐analyses based on follow‐up timeframe and omitted PSSP intervention arms (Pistone et al., 2019).

### Limitations

Meta‐regression findings warrant caution due to small numbers of effects across moderator categories, which can increase sampling error in regression weights (Schmidt, 2017). Lack of necessary outcome data resulted in the exclusion of 3/15 screened studies. Additionally, school characteristics (e.g., school type and school size) were under‐reported across included CRT studies. Considering that school‐level variables explained a small but significant amount of variability in youth mental health among 26,885 students (Ford et al., 2021), school‐level characteristics should be fully reported in studies that examine interventions aiming to reduce STBs. Although publication bias appears to not be an issue, the effectiveness of PSSP may be overestimated in the present meta‐analysis if nonsignificant SI and SA outcomes were not reported by studies evaluating interventions eligible for inclusion, which particularly may be an issue for interventions that target SA and SI with other health and well‐being outcomes. Therefore, full reporting and availability of data should be prioritised in PSSP intervention research.

The combined weight of trials from the same study for the crude composite SA estimate was 69.81%, and heterogeneity between trials from the same study and trials from independent studies was moderate at 51.20%. However, the combined weight of trials from the same study for the adjusted composite SA estimate was 55.61%, and between‐study heterogeneity = 0%, which suggests that trials from the same study did not greatly influence the adjusted composite SA effects. Collectively, we recommend that interpretations regarding the effectiveness of PSSP on SA herein be based on both crude and adjusted estimates or conservatively based only on the adjusted estimate.

Only one study (Wasserman et al., 2015) reported measuring deaths by suicide. Although many PSSP interventions proximally aim to target STBs due to the challenges to evaluating interventions targeting suicide (Balaguru et al., 2012), understanding the impact of PSSP on deaths by suicide is essential. Only one study included young people actively involved in delivering interventions (Wyman et al., 2010), despite emerging research highlighting the importance of youth involvement in prevention of youth suicide (Thorn et al., 2020; Trinh & Goebert, 2020). No studies were conducted in Africa or South America. More replication trials are needed to clarify which specific interventions are most effective and where; of the retained studies, the SOS programme was replicated across multiple trials based in North America, and the YAM, QPR, and Profscreen interventions were examined across 10 European Union countries as part of the SEYLE study. Replication is needed across various contexts, particularly given the cultural variation of STBs (Page et al., 2013), and the variability in schooling contexts across jurisdictions.

## Conclusions

The present findings contribute to the PSSP evidence‐base by supporting PSSP targeting adolescent STBs as both primary intervention outcomes and with other health and well‐being outcomes as a clinically relevant approach to effectively target SA and SI. Moderator analyses preliminarily support PSSP interventions of ≤1‐week duration, involving multiple stakeholders and with a 12‐month follow‐up as particularly effective for reducing SA, but given the small number of included trials conclusions warrant clarification by future research. Findings highlight the need for greater consideration of how intervention and contextual factors impact PSSP effectiveness. Findings should be of interest to researchers, clinicians, educators, and policymakers.

## Supporting information


**Figure S1.** Forest plot of crude suicidal ideation odd ratios.

**Figure S2.** Forest plot of crude suicide attempts odd ratios.

**Figure S3.** Forest plot of adjusted suicidal ideation odd ratios.

**Figure S4.** Forest plot of adjusted suicide attempts odd ratios.

**Table S1.** PRISMA 2020 checklist.
**Table S2.** Complete searches.
**Table S3.** Summary of PICOs, intervention, and contextual factors for studies measuring suicidal ideation and/or attempts.
**Table S4.** Study characteristics adjustments.
**Table S5.** Meta‐analysis and univariate meta‐regressions for suicide attempt crude log ORs (*k* = 13) (inclusion of Shinde et al 2020 teacher‐led trial).
**Table S6.** Summary of univariate analyses of moderators for crude suicide attempt log odds ratio.
**Table S7.** Summary of univariate analyses of moderators for adjusted suicide attempt log odds ratio.
**Table S8.** Amendments to protocol.

## References

[jcpp13598-bib-0001] Ahern, S. , Burke, L.‐A. , McElroy, B. , Corcoran, P. , McMahon, E.M. , Keeley, H. , … & Wasserman, D. (2018). A cost‐effectiveness analysis of school‐based suicide prevention programmes. European Child & Adolescent Psychiatry, 27(10), 1295–1304. 29442231 10.1007/s00787-018-1120-5

[jcpp13598-bib-0002] Altman, D.G. , & Bland, J.M. (2011). How to obtain the P value from a confidence interval. BMJ, 343, d2304. 22803193 10.1136/bmj.d2304

[jcpp13598-bib-0003] Aseltine, R.H., Jr , James, A. , Schilling, E.A. , & Glanovsky, J. (2007). Evaluating the SOS suicide prevention program: A replication and extension. BMC Public Health, 7, 161. 17640366 10.1186/1471-2458-7-161 PMC1941734

[jcpp13598-bib-0004] Balaguru, V. , Sharma, J. , & Waheed, W. (2012). Understanding the effectiveness of school‐based interventions to prevent suicide: A realist review. Child and Adolescent Mental Health, 18(3), 131–139. 32847255 10.1111/j.1475-3588.2012.00668.x

[jcpp13598-bib-0005] Borenstein, M. , Hedges, L.V. , Higgins, J.P. , & Rothstein, H.R. (2011). Introduction to meta‐analysis. West Sussex, UK: John Wiley and Sons.

[jcpp13598-bib-0006] Brann, K.L. , Baker, D. , Smith‐Millman, M.K. , Watt, S.J. , & DiOrio, C. (2021). A meta‐analysis of suicide prevention programs for school‐aged youth. Children and Youth Services Review, 121, 105826.

[jcpp13598-bib-0007] Breet, E. , Matooane, M. , Tomlinson, M. , & Bantjes, J. (2021). Systematic review and narrative synthesis of suicide prevention in high‐schools and universities: A research agenda for evidence‐based practice. BMC Public Health, 21(1), 1116. 34112141 10.1186/s12889-021-11124-w PMC8194002

[jcpp13598-bib-0008] Burns, P.B. , Rohrich, R.J. , & Chung, K.C. (2011). The levels of evidence and their role in evidence‐based medicine. Plastic and Reconstructive Surgery, 128(1), 305–310. 21701348 10.1097/PRS.0b013e318219c171 PMC3124652

[jcpp13598-bib-0009] Centers for Disease Control and Prevention (2020). Leading causes of death reports, 1981 ‐ 2018. https://webappa.cdc.gov/sasweb/ncipc/leadcause.html

[jcpp13598-bib-0010] Cohen, J. (1960). A coefficient of agreement for nominal scales. Educational and Psychological Measurement, 20(1), 37–46.

[jcpp13598-bib-0011] Cook, R.D. , & Weisberg, S. (1982). Residuals and influence in regression. New York, NY: Chapman and Hall.

[jcpp13598-bib-0012] Domitrovich, C.E. , Bradshaw, C.P. , Poduska, J.M. , Hoagwood, K. , Buckley, J.A. , Olin, S. , … & Ialongo, N.S. (2008). Maximizing the implementation quality of evidence‐based preventive interventions in schools: A conceptual framework. Advances in School Mental Health Promotion, 3(1), 6–28. 10.1080/1754730x.2008.9715730 PMC4865398 27182282

[jcpp13598-bib-0013] Donner, A. , & Klar, N. (2002). Issues in the meta‐analysis of cluster randomized trials. Statistics in Medicine, 21(19), 2971–2980. 12325113 10.1002/sim.1301

[jcpp13598-bib-0014] Egger, M. , Smith, G.D. , Schneider, M. , & Minder, C. (1997). Bias in meta‐analysis detected by a simple, graphical test. BMJ, 315(7109), 629–634. 9310563 10.1136/bmj.315.7109.629 PMC2127453

[jcpp13598-bib-0015] Eldridge, S. , Campbell, M. , Campbell, M. , Drahota‐Towns, A. , Giraudeau, B. , Higgins, J. , … & Siegfried, N. (2016). Revised Cochrane risk of bias tool for randomized trials (RoB 2.0) Additional considerations for cluster‐randomized trials. https://sites.google.com/site/riskofbiastool/welcome/rob‐2‐0‐tool

[jcpp13598-bib-0016] Fekkes, M. , van de Sande, M.E. , Gravesteijn, J.C. , Pannebakker, F.D. , Buijs, G.J. , Diekstra, R. , & Kocken, P.L. (2016). Effects of the Dutch Skills for Life program on the health behavior, bullying, and suicidal ideation of secondary school students. Health Education, 116(1), 2–15.

[jcpp13598-bib-0017] Fernandez‐Castilla, B. , Jamshidi, L. , Declercq, L. , Beretvas, S.N. , Onghena, P. , & Van den Noortgate, W. (2020). The application of meta‐analytic (multi‐level) models with multiple random effects: A systematic review. Behavior Research Methods, 52, 2031–2052. 32162276 10.3758/s13428-020-01373-9

[jcpp13598-bib-0018] Flay, B.R. , Biglan, A. , Boruch, R.F. , Castro, F.G. , Gottfredson, D. , Kellam, S. , … & Ji, P. (2005). Standards of evidence: Criteria for efficacy, effectiveness and dissemination. Prevention Science, 6(3), 151–175. 16365954 10.1007/s11121-005-5553-y

[jcpp13598-bib-0070] Ford, T. , Degli Esposti, M. , Crane, C. , Taylor, L. , Montero‐Marín, J. , Blakemore, S.‐J. , … & Wainman, B. (2021). The role of schools in early adolescents’ mental health: findings from the MYRIAD study. Journal of the American Academy of Child & Adolescent Psychiatry, 60(12), 1467–1478. 10.1016/j.jaac.2021.02.016 33677037 PMC8669152

[jcpp13598-bib-0019] Gijzen, M.W.M. , Rasing, S.P.A. , Creemers, D.H.M. , Engels, R.C.M.E. , & Smit, F. (2022). Effectiveness of school‐based preventive programs in suicidal thoughts and behaviors: A meta‐analysis. Journal of Affective Disorders, 298, 408–420. 34728296 10.1016/j.jad.2021.10.062

[jcpp13598-bib-0020] Goldsmith, S.K. (2002). Reducing suicide a national imperative. Washington, DC: National Academies Press. 25057611

[jcpp13598-bib-0021] Gould, M.S. , Marrocco, F.A. , Kleinman, M. , Thomas, J.G. , Mostkoff, K. , Cote, J. , & Davies, M. (2005). Evaluating iatrogenic risk of youth suicide screening programs: A randomized controlled trial. JAMA, 293(13), 1635–1643. 15811983 10.1001/jama.293.13.1635

[jcpp13598-bib-0022] Harrer, M. , Cuijpers, P. , Furukawa, T.A. , & Ebert, D.D. (2019). Doing meta‐analysis in R: A hands‐on guide. https://bookdown.org/mathiasharrer/doing_meta_analysis_in_r/

[jcpp13598-bib-0023] Higgins, J.P.T. , Altman, D.G. , Gotzsche, P.C. , Juni, P. , Moher, D. , Oxman, A.D. , … & Sterne, J.A.C. (2011). The Cochrane Collaboration's tool for assessing risk of bias in randomised trials. BMJ, 343, d5928. 22008217 10.1136/bmj.d5928 PMC3196245

[jcpp13598-bib-0024] Higgins, J.P. , & Thompson, S.G. (2002). Quantifying heterogeneity in a meta‐analysis. Statistics in Medicine, 21(11), 1539–1558. 12111919 10.1002/sim.1186

[jcpp13598-bib-0025] Hofstra, E. , van Nieuwenhuizen, C. , Bakker, M. , Özgül, D. , Elfeddali, I. , de Jong, S.J. , & van der Feltz‐Cornelis, C.M. (2020). Effectiveness of suicide prevention interventions: A systematic review and meta‐analysis. General Hospital Psychiatry, 63, 127–140. 31078311 10.1016/j.genhosppsych.2019.04.011

[jcpp13598-bib-0026] Jozefowicz‐Simbeni, D.M.H. (2008). An ecological and developmental perspective on dropout risk factors in early adolescence: Role of school social workers in dropout prevention efforts. Children & Schools, 30(1), 49–62.

[jcpp13598-bib-0027] Kapur, N. , & Gask, L. (2009). Introduction to suicide and self‐harm. Psychiatry, 8(7), 233–236.

[jcpp13598-bib-0028] Large, M.M. , Ryan, C.J. , Carter, G. , & Kapur, N. (2017). Can we usefully stratify patients according to suicide risk? BMJ, 359, j4627. 29042363 10.1136/bmj.j4627

[jcpp13598-bib-0029] Matsubayashi, T. , & Ueda, M. (2011). The effect of national suicide prevention programs on suicide rates in 21 OECD nations. Social Science & Medicine, 73(9), 1395–1400. 21940085 10.1016/j.socscimed.2011.08.022

[jcpp13598-bib-0030] Mendes, D. , Alves, C. , & Batel‐Marques, F. (2017). Number needed to treat (NNT) in clinical literature: An appraisal. BMC Medicine, 15(1), 112. 28571585 10.1186/s12916-017-0875-8 PMC5455127

[jcpp13598-bib-0068] Methley, A.M. , Campbell, S. , Chew‐Graham, C. , McNally, R. , Cheraghi‐Sohi, S. (2014). PICO, PICOS and SPIDER: a comparison study of specificity and sensitivity in three search tools for qualitative systematic reviews. BMC Health Services Research, 14(1), 579. 10.1186/s12913-014-0579-0 25413154 PMC4310146

[jcpp13598-bib-0031] Miller, D.N. , Eckert, T.L. , & Mazza, J.J. (2009). Suicide prevention programs in the schools: A review and public health perspective. School Psychology Review, 38(2), 168–188.

[jcpp13598-bib-0032] Miller, I.W. , Camargo, C.A. , Arias, S.A. , Sullivan, A.F. , Allen, M.H. , Goldstein, A.B. , … & Boudreaux, E.D. (2017). Suicide prevention in an emergency department population: The ED‐SAFE study. JAMA Psychiatry, 74(6), 563–570. 28456130 10.1001/jamapsychiatry.2017.0678 PMC5539839

[jcpp13598-bib-0033] Mittman, B.S. (2004). Creating the evidence base for quality improvement collaboratives. Annals of Internal Medicine, 140(11), 897–901. 15172904 10.7326/0003-4819-140-11-200406010-00011

[jcpp13598-bib-0069] Nock, M.K. , Green, J.G. , Hwang, I. , McLaughlin, K.A. , Sampson, N.A. , Zaslavsky, A.M. , & Kessler, R.C. (2013). Prevalence, correlates, and treatment of lifetime suicidal behavior among adolescents: results from the National Comorbidity Survey Replication Adolescent Supplement. JAMA Psychiatry, 70(3), 300–310. https://jamanetwork.com/journals/jamapsychiatry/article‐abstract/1555602 23303463 10.1001/2013.jamapsychiatry.55 PMC3886236

[jcpp13598-bib-0034] O’Leary‐Barrett, M. , Topper, L. , Al‐Khudhairy, N. , Pihl, R.O. , Castellanos‐Ryan, N. , Mackie, C.J. , & Conrod, P.J. (2013). Two‐year impact of personality‐targeted, teacher‐delivered interventions on youth internalizing and externalizing problems: A cluster‐randomized trial. The Journal of the American Academy of Child and Adolescent Psychiatry, 52(9), 911–920. 23972693 10.1016/j.jaac.2013.05.020

[jcpp13598-bib-0035] Orbach, I. , & Hanna, B.‐J. (1993). The impact of a suicide prevention program for adolescents on suicidal tendencies, hopelessness, ego identity, and coping. Suicide and LifeThreatening Behavior, 23(2), 120–129. 8342211

[jcpp13598-bib-0036] Ougrin, D. , Tranah, T. , Stahl, D. , Moran, P. , & Rosenbaum Asarnow, J. (2015). Therapeutic interventions for suicide attempts and self‐harm in adolescents: Systematic review and meta‐analysis. Journal of the American Academy of Child and Adolescent Psychiatry, 54(2), 97–107. 25617250 10.1016/j.jaac.2014.10.009

[jcpp13598-bib-0037] Ouzzani, M. , Hammady, H. , Fedorowicz, Z. , & Elmagarmid, A. (2016). Rayyan — A web and mobile app for systematic reviews. Systematic Reviews, 5(1), 1–10. 27919275 10.1186/s13643-016-0384-4 PMC5139140

[jcpp13598-bib-0038] Page, M.J. , McKenzie, J.E. , Bossuyt, P.M. , Boutron, I. , Hoffmann, T.C. , Mulrow, C.D. , … & Moher, D. (2021). The PRISMA 2020 statement: An updated guideline for reporting systematic reviews. BMJ, 372, n71. 33782057 10.1136/bmj.n71 PMC8005924

[jcpp13598-bib-0039] Page, R.M. , Saumweber, J. , Hall, P.C. , Crookston, B.T. , & West, J.H. (2013). Multi‐country, cross‐national comparison of youth suicide ideation: Findings from Global School‐based Health Surveys. School Psychology International, 34(5), 540–555.

[jcpp13598-bib-0040] Perry, Y. , Petrie, K. , Buckley, H. , Cavanagh, L. , Clarke, D. , Winslade, M. , … & Christensen, H. (2014). Effects of a classroom‐based educational resource on adolescent mental health literacy: A cluster randomized controlled trial. Journal of Adolesence, 37(7), 1143–1151. 10.1016/j.adolescence.2014.08.001 25151646

[jcpp13598-bib-0041] Perry, Y. , Werner‐Seidler, A. , Calear, A. , Mackinnon, A. , King, C. , Scott, J. , … & Batterham, P.J. (2017). Preventing depression in final year secondary students: School‐based randomized controlled trial. Journal of Medical Internet Research, 19(11), e369. 29097357 10.2196/jmir.8241 PMC5691241

[jcpp13598-bib-0042] Pistone, I. , Beckman, U. , Eriksson, E. , Lagerlof, H. , & Sager, M. (2019). The effects of educational interventions on suicide: A systematic review and meta‐analysis. International Journal of Social Psychiatry, 65(5), 399–412. 31159627 10.1177/0020764019852655

[jcpp13598-bib-0043] Poppelaars, M. , Tak, Y.R. , Lichtwarck‐Aschoff, A. , Engels, R.C.M.E. , Lobel, A. , Merry, S.N. , … & Granic, I. (2016). A randomized controlled trial comparing two cognitive‐behavioral programs for adolescent girls with subclinical depression: A school‐based program (Op Volle Kracht) and a computerized program (SPARX). Behavior Research Therapy, 80, 33–42. 10.1016/j.brat.2016.03.005 27019280

[jcpp13598-bib-0044] Randell, B.P. , Eggert, L.L. , & Pike, K.C. (2001). Immediate post intervention effects of two brief youth suicide prevention interventions. Suicide and Life‐Threatening Behavior, 31(1), 41–61. 11326768 10.1521/suli.31.1.41.21308

[jcpp13598-bib-0045] Restore @ National Centre for Research Methods (2011). 4.2 An introduction to odds, odds ratios and exponents Using statistical regression methods in education research 2021. https://www.restore.ac.uk/srme/www/fac/soc/wie/research‐new/srme/modules/mod4/2/index.html

[jcpp13598-bib-0046] Robinson, J.O. , Bailey, E. , Witt, K. , Stefanac, N. , Milner, A. , Currier, D. , … & Hetrick, S. (2018). What works in youth suicide prevention? A systematic review and meta‐analysis. E Clinical Medicine, 4–5, 52–91. 10.1016/j.eclinm.2018.10.004 PMC6537558 31193651

[jcpp13598-bib-0047] Schilling, E.A. , Aseltine, R.H., Jr , & James, A. (2016). The SOS suicide prevention program: Further evidence of efficacy and effectiveness. Prevention Science, 17(2), 157–166. 26314868 10.1007/s11121-015-0594-3

[jcpp13598-bib-0048] Schilling, E.A. , Lawless, M. , Buchanan, L. , & Aseltine, R.H., Jr (2014). "Signs of Suicide" shows promise as a middle school suicide prevention program. Suicide and Life Threatening Behavior, 44(6), 653–667. 24796660 10.1111/sltb.12097

[jcpp13598-bib-0049] Schmidt, F.L. (2017). Statistical and measurement pitfalls in the use of meta‐regression in meta‐analysis. Career Development International, 22(5), 469–476.

[jcpp13598-bib-0050] Schünemann, H. , Brożek, J. , Guyatt, G. , & Oxman, A. (2013). GRADE handbook for grading quality of evidence and strength of recommendations. The GRADE Working Group. https://gdt.gradepro.org/app/handbook/handbook.html

[jcpp13598-bib-0051] Schwarzer, G. (2007). Meta: An R package for meta‐analysis. R News, 7(3), 40–45.

[jcpp13598-bib-0052] Shinde, S. , Weiss, H.A. , Khandeparkar, P. , Pereira, B. , Sharma, A. , Gupta, R. , … & Patel, V. (2020). A multicomponent secondary school health promotion intervention and adolescent health: An extension of the SEHER cluster randomised controlled trial in Bihar, India. PLoS Medicine, 17(2), e1003021. 32045409 10.1371/journal.pmed.1003021 PMC7012396

[jcpp13598-bib-0053] Surgenor, P.W. , Quinn, P. , & Hughes, C. (2016). Ten recommendations for effective school‐based, adolescent, suicide prevention programs. School Mental Health, 8(4), 413–424.

[jcpp13598-bib-0054] Tang, J.J. , Yu, Y. , Wilcox, H.C. , Kang, C. , Wang, K. , Wang, C. , … & Chen, R. (2020). Global risks of suicidal behaviours and being bullied and their association in adolescents: School‐based health survey in 83 countries. EClinicalMedicine, 19, 100253. 32140671 10.1016/j.eclinm.2019.100253 PMC7046520

[jcpp13598-bib-0055] Tarrier, N. , Taylor, K. , & Gooding, P. (2008). Cognitive‐behavioral interventions to reduce suicide behavior. Behavior Modification, 32(1), 77–108. 18096973 10.1177/0145445507304728

[jcpp13598-bib-0056] Thorn, P. , Hill, N.T.M. , Lamblin, M. , Teh, Z. , Battersby‐Coulter, R. , Rice, S. , … & Robinson, J.O. (2020). Developing a suicide prevention social media campaign with young people (the #Chatsafe Project): co‐design approach. JMIR Ment Health, 7(5), e17520. 32391800 10.2196/17520 PMC7248803

[jcpp13598-bib-0057] Trinh, T. , & Goebert, D. (2020). Youth voice in suicide prevention in Hawai'i. Hawai'i Journal of Health & Social Welfare, 79(5 Suppl 1), 71–75. PMC7260881 32490389

[jcpp13598-bib-0058] van Vuuren, C.L. , van der Wal, M.F. , Cuijpers, P. , & Chinapaw, M.J.M. (2020). Are suicidal thoughts and behaviors a temporary phenomenon in early adolescence? Crisis, 42, 78–81. 32228038 10.1027/0227-5910/a000680 PMC8208294

[jcpp13598-bib-0059] Viechtbauer, W. (2010). Conducting meta‐analyses in R with the metafor package. Journal of Statistical Software, 36(3), 1–48.

[jcpp13598-bib-0060] Viechtbauer Wolfgang , Cheung Mike, W.‐L. (2010). Outlier and influence diagnostics for meta‐analysis. Research Synthesis Methods, 1, (2), 112. –125. 10.1002/jrsm.11 26061377

[jcpp13598-bib-0061] Vieland, V. , Whittle, B. , Garland, A. , Hicks, R. , & Shaffer, D. (1991). The impact of curriculum‐based suicide prevention programs for teenagers: An I8‐month follow‐up. Journal of the American Academy of Child & Adolescent Psychiatry, 30(4), 588–596. 1938799

[jcpp13598-bib-0062] Wasserman, D. , Hoven, C.W. , Wasserman, C. , Wall, M. , Eisenberg, R. , Hadlaczky, G. , … & Carli, V. (2015). School‐based suicide prevention programmes: The SEYLE cluster‐randomised, controlled trial. The Lancet, 385(9977), 1536–1544. 10.1016/S0140-6736(14)61213-7 25579833

[jcpp13598-bib-0063] White, J. , Morris, J. , & Hinbest, J. (2012). Collaborative knowledge‐making in the everyday practice of youth suicide prevention education. International Journal of Qualitative Studies in Education, 25(3), 339–355.

[jcpp13598-bib-0064] World Health Organisation Geneva: World Health Organisation. https://apps.who.int/iris/handle/10665/279765

[jcpp13598-bib-0065] World Health Organisation (2021). Suicide. https://www.who.int/news‐room/fact‐sheets/detail/suicide

[jcpp13598-bib-0066] Wyman, P.A. (2014). Developmental approach to prevent adolescent suicides: Research pathways to effective upstream preventive interventions. American Journal of Preventive Medicine, 47(3 Suppl 2), S251–S256. 25145747 10.1016/j.amepre.2014.05.039 PMC4143775

[jcpp13598-bib-0067] Wyman, P.A. , Brown, C.H. , LoMurray, M. , Schmeelk‐Cone, K. , Petrova, M. , Yu, Q. , … & Wang, W. (2010). An outcome evaluation of the Sources of Strength suicide prevention program delivered by adolescent peer leaders in high schools. American Journal of Public Health, 100(9), 1653–1661. 20634440 10.2105/AJPH.2009.190025 PMC2920978

