# Supplementary figures and images for: Research Review: The effect of school‐based suicide prevention on suicidal ideation and suicide attempts and the role of intervention and contextual factors among adolescents: a meta‐analysis and meta‐regression
Source: J Child Psychol Psychiatry. 2022 Mar 15;63(8):836–45. doi: 10.1111/jcpp.13598 (PMC9544521; doi:10.1111/jcpp.13598)

# Forest plot of crude suicidal ideation odd ratios

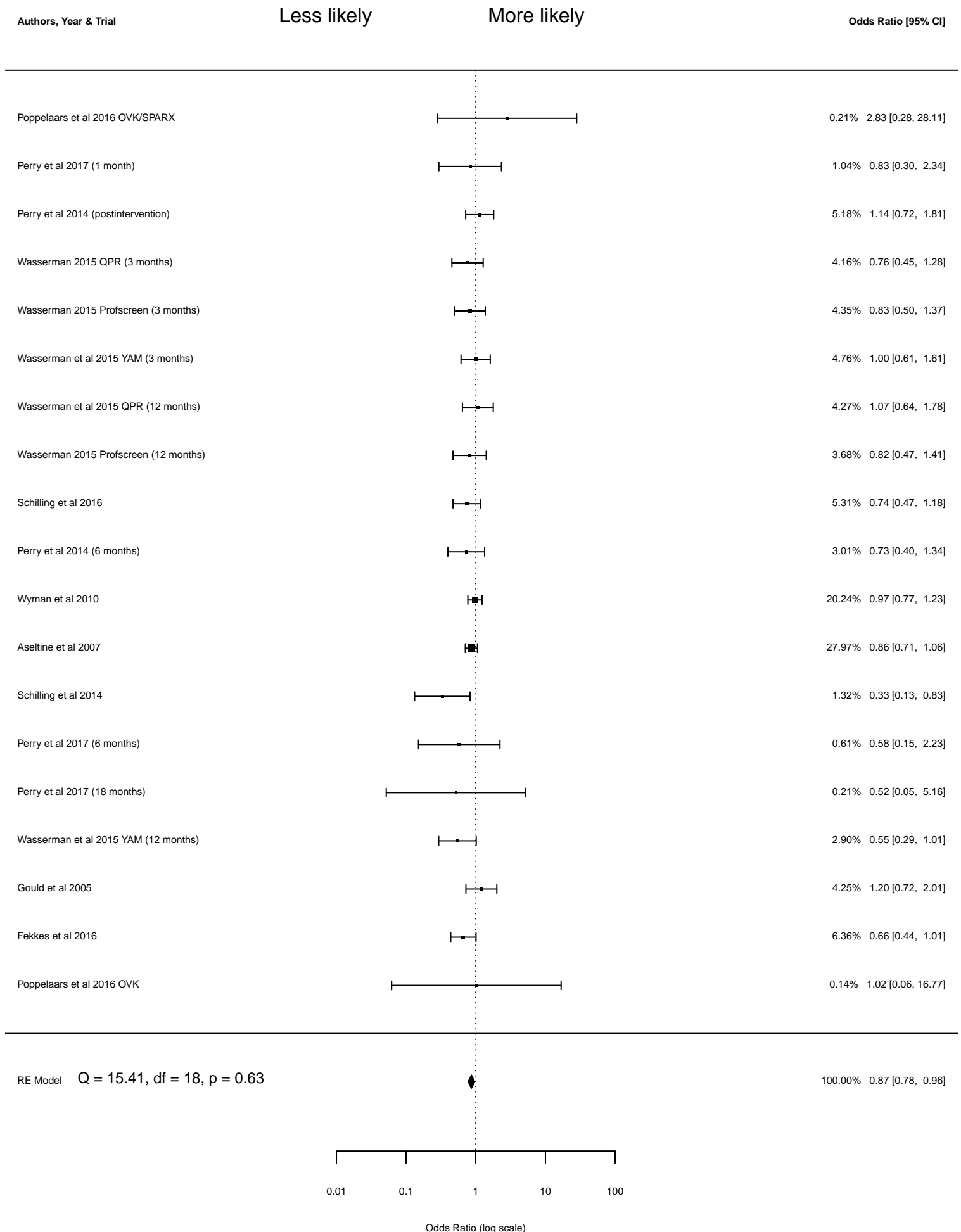

Supplement: Supplementary file 1 — Figure S1. Forest plot of crude suicidal ideation odd ratios. [file JCPP-63-836-s002.pdf]

# Forest plot of crude suicide attempts odd ratios

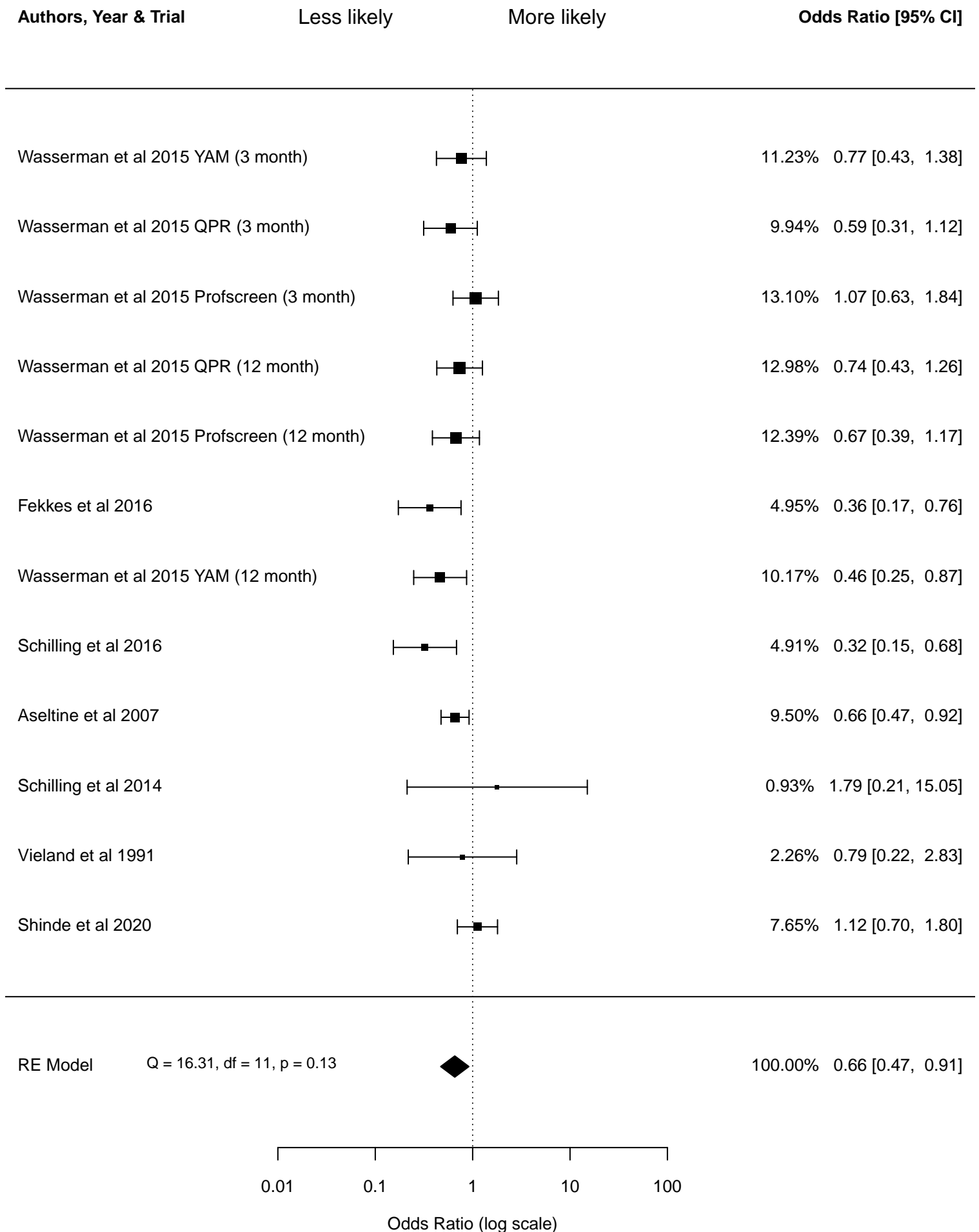

Supplement: Supplementary file 2 — Figure S2. Forest plot of crude suicide attempts odd ratios. [file JCPP-63-836-s003.pdf]

# Forest plot of adjusted suicidal ideation odd ratios

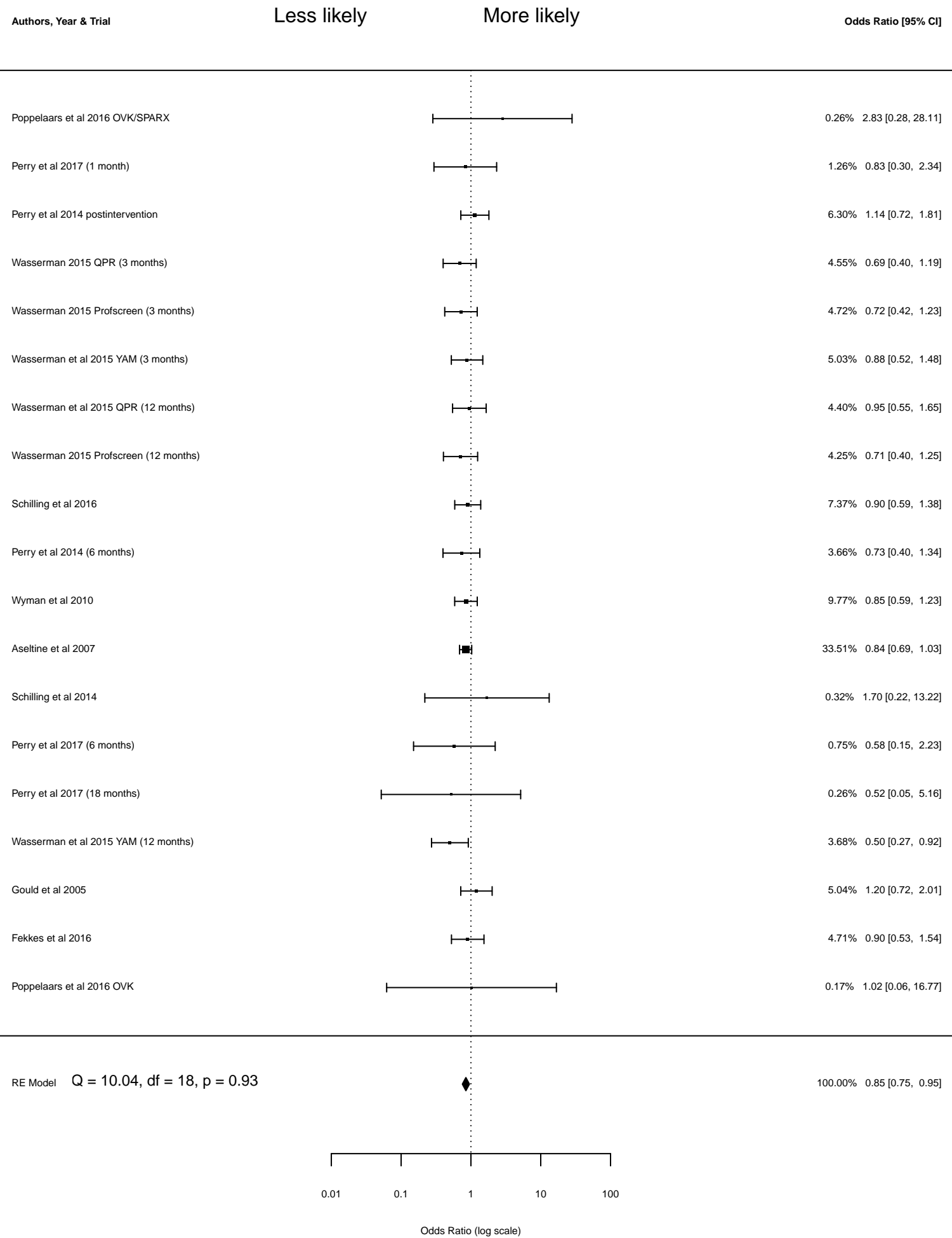

Supplement: Supplementary file 3 — Figure S3. Forest plot of adjusted suicidal ideation odd ratios. [file JCPP-63-836-s005.pdf]

### Forest plot of adjusted suicide attempts odd ratios

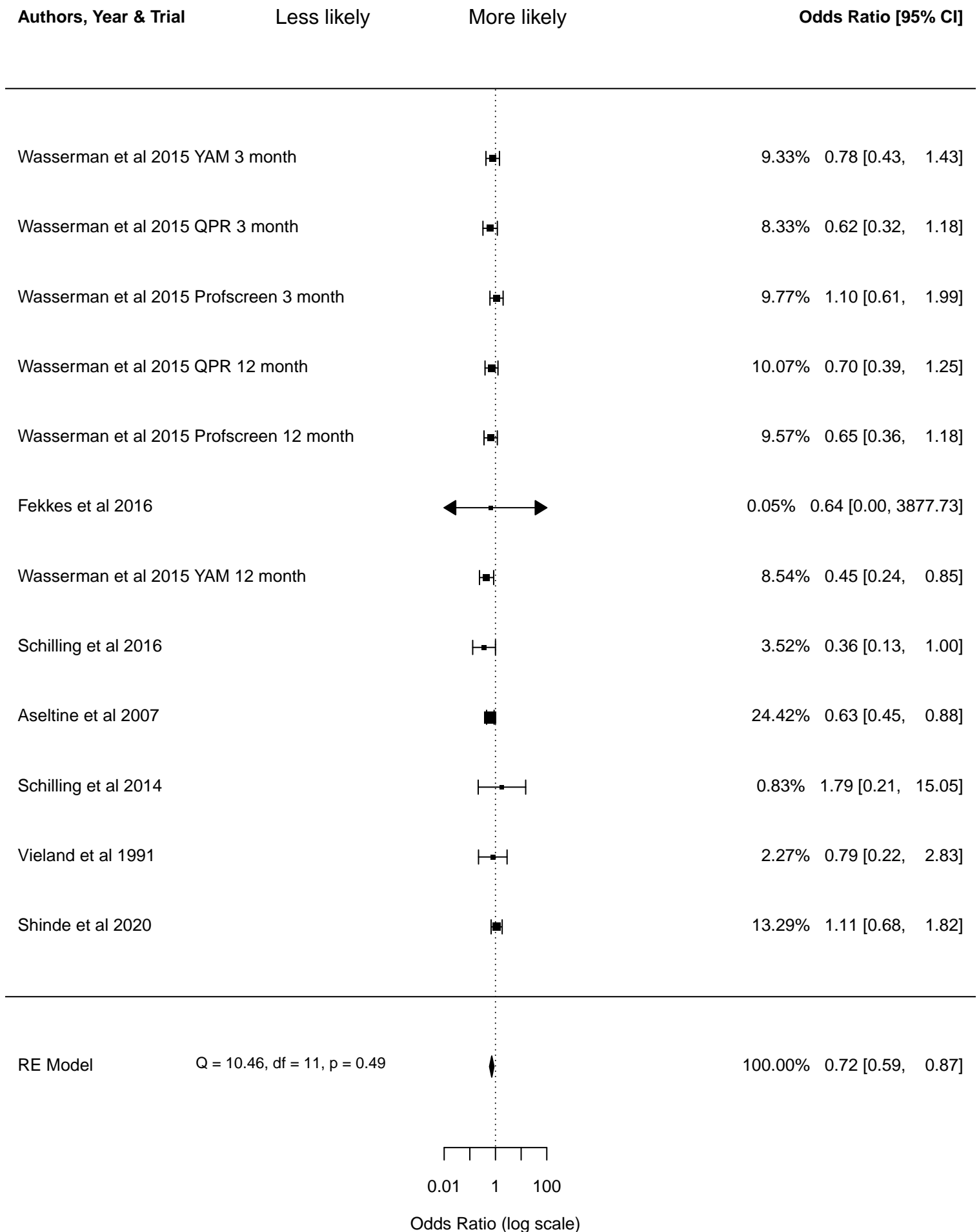

Supplement: Supplementary file 4 — Figure S4. Forest plot of adjusted suicide attempts odd ratios. [file JCPP-63-836-s004.pdf]
