# Supplementary material for: Research Review: The effect of school‐based suicide prevention on suicidal ideation and suicide attempts and the role of intervention and contextual factors among adolescents: a meta‐analysis and meta‐regression
Source: J Child Psychol Psychiatry. 2022 Mar 15;63(8):836–45. doi: 10.1111/jcpp.13598 (PMC9544521; doi:10.1111/jcpp.13598)
Supplement: Supplementary file 5 — Table S1. PRISMA 2020 checklist. Table S2. Complete searches. Table S3. Summary of PICOs, intervention, and contextual factors for studies measuring suicidal ideation and/or attempts. Table S4. Study characteristics adjustments. Table S5. Meta‐analysis and univariate meta‐regressions for suicide attempt crude log ORs (k = 13) (inclusion of Shinde et al 2020 teacher‐led trial). Table S6. Summary of univariate analyses of moderators for crude suicide attempt log odds ratio. Table S7. Summary of univariate analyses of moderators for adjusted suicide attempt log odds ratio. Table S8. Amendments to protocol. [file JCPP-63-836-s001.docx]

**Supporting Information**

**Table S1. PRISMA 2020 Checklist**

| **Section and Topic** | **Item #** | **Checklist item** | **Location where item is reported** |
| --- | --- | --- | --- |
| **TITLE** | | |  |
| Title | 1 | Identify the report as a systematic review. | Page 1 |
| **ABSTRACT** | | |  |
| Abstract | 2 | See the PRISMA 2020 for Abstracts checklist. | Page Abstract |
| **INTRODUCTION** | | |  |
| Rationale | 3 | Describe the rationale for the review in the context of existing knowledge. | Pages 2-4 |
| Objectives | 4 | Provide an explicit statement of the objective(s) or question(s) the review addresses. | Page 5 |
| **METHODS** | | |  |
| Eligibility criteria | 5 | Specify the inclusion and exclusion criteria for the review and how studies were grouped for the syntheses. | Pages 5-6 |
| Information sources | 6 | Specify all databases, registers, websites, organisations, reference lists and other sources searched or consulted to identify studies. Specify the date when each source was last searched or consulted. | Page 5 |
| Search strategy | 7 | Present the full search strategies for all databases, registers and websites, including any filters and limits used. | Table S2 |
| Selection process | 8 | Specify the methods used to decide whether a study met the inclusion criteria of the review, including how many reviewers screened each record and each report retrieved, whether they worked independently, and if applicable, details of automation tools used in the process. | Page 5 |
| Data collection process | 9 | Specify the methods used to collect data from reports, including how many reviewers collected data from each report, whether they worked independently, any processes for obtaining or confirming data from study investigators, and if applicable, details of automation tools used in the process. | Page 6 |
| Data items | 10a | List and define all outcomes for which data were sought. Specify whether all results that were compatible with each outcome domain in each study were sought (e.g. for all measures, time points, analyses), and if not, the methods used to decide which results to collect. | Pages 6-7 |
|  | 10b | List and define all other variables for which data were sought (e.g. participant and intervention characteristics, funding sources). Describe any assumptions made about any missing or unclear information. | Pages 6-8 Table S3 |
| Study risk of bias assessment | 11 | Specify the methods used to assess risk of bias in the included studies, including details of the tool(s) used, how many reviewers assessed each study and whether they worked independently, and if applicable, details of automation tools used in the process. | Page 6 |
| Effect measures | 12 | Specify for each outcome the effect measure(s) (e.g. risk ratio, mean difference) used in the synthesis or presentation of results. | Page 6 |
| Synthesis methods | 13a | Describe the processes used to decide which studies were eligible for each synthesis (e.g. tabulating the study intervention characteristics and comparing against the planned groups for each synthesis (item #5)). | Table S3, pages 6 & 8 |
|  | 13b | Describe any methods required to prepare the data for presentation or synthesis, such as handling of missing summary statistics, or data conversions. | Pages 6-7, 8 |
|  | 13c | Describe any methods used to tabulate or visually display results of individual studies and syntheses. | Pages 6, 8 |
|  | 13d | Describe any methods used to synthesize results and provide a rationale for the choice(s). If meta-analysis was performed, describe the model(s), method(s) to identify the presence and extent of statistical heterogeneity, and software package(s) used. | Pages 6-8 |
|  | 13e | Describe any methods used to explore possible causes of heterogeneity among study results (e.g. subgroup analysis, meta-regression). | Pages 7-8 |
|  | 13f | Describe any sensitivity analyses conducted to assess robustness of the synthesized results. | Page 8 |
| Reporting bias assessment | 14 | Describe any methods used to assess risk of bias due to missing results in a synthesis (arising from reporting biases). | Page 8-9 |
| Certainty assessment | 15 | Describe any methods used to assess certainty (or confidence) in the body of evidence for an outcome. | Pages 7-9 |
| **RESULTS** | | |  |
| Study selection | 16a | Describe the results of the search and selection process, from the number of records identified in the search to the number of studies included in the review, ideally using a flow diagram. | Figure1 |
|  | 16b | Cite studies that might appear to meet the inclusion criteria, but which were excluded, and explain why they were excluded. | Pages 6 & 9 |
| Study characteristics | 17 | Cite each included study and present its characteristics. | Table S3  Pages 9-10 |
| Risk of bias in studies | 18 | Present assessments of risk of bias for each included study. | Table S3 |
| Results of individual studies | 19 | For all outcomes, present, for each study: (a) summary statistics for each group (where appropriate) and (b) an effect estimate and its precision (e.g. confidence/credible interval), ideally using structured tables or plots. | FigureS1-FiguresS4 & pages 10-11 |
| Results of syntheses | 20a | For each synthesis, briefly summarise the characteristics and risk of bias among contributing studies. | Page 10, Table S3 |
|  | 20b | Present results of all statistical syntheses conducted. If meta-analysis was done, present for each the summary estimate and its precision (e.g. confidence/credible interval) and measures of statistical heterogeneity. If comparing groups, describe the direction of the effect. | Page 10-11  Table S5  FigureS1-Figures4 |
|  | 20c | Present results of all investigations of possible causes of heterogeneity among study results. | Table S5-Table S7, page 11 |
|  | 20d | Present results of all sensitivity analyses conducted to assess the robustness of the synthesized results. | Pages 10-11 |
| Reporting biases | 21 | Present assessments of risk of bias due to missing results (arising from reporting biases) for each synthesis assessed. | Page 11 |
| Certainty of evidence | 22 | Present assessments of certainty (or confidence) in the body of evidence for each outcome assessed. | Pages 10-11 |
| **DISCUSSION** | | |  |
| Discussion | 23a | Provide a general interpretation of the results in the context of other evidence. | Pages 11-16 |
|  | 23b | Discuss any limitations of the evidence included in the review. | Pages 16-17 |
|  | 23c | Discuss any limitations of the review processes used. | Pages 16-17 |
|  | 23d | Discuss implications of the results for practice, policy, and future research. | Pages 13-14, 17-18 |
| **OTHER INFORMATION** | | |  |
| Registration and protocol | 24a | Provide registration information for the review, including register name and registration number, or state that the review was not registered. | Page 5 |
|  | 24b | Indicate where the review protocol can be accessed, or state that a protocol was not prepared. | Page 5 |
|  | 24c | Describe and explain any amendments to information provided at registration or in the protocol. | Table S8 |
| Support | 25 | Describe sources of financial or non-financial support for the review, and the role of the funders or sponsors in the review. | Page 1 |
| Competing interests | 26 | Declare any competing interests of review authors. | Page 1 |
| Availability of data, code and other materials | 27 | Report which of the following are publicly available and where they can be found: template data collection forms; data extracted from included studies; data used for all analyses; analytic code; any other materials used in the review. | Table S3 |

*From:*  Page MJ, McKenzie JE, Bossuyt PM, Boutron I, Hoffmann TC, Mulrow CD, et al. The PRISMA 2020 statement: an updated guideline for reporting systematic reviews. BMJ 2021;372:n71. doi: 10.1136/bmj.n71

For more information, visit: <http://www.prisma-statement.org/>

**Table S2. Complete searches**

| **Databases/registers** | **Search strings** |
| --- | --- |
| Aggregate electronic database EBSCO host (containing Education Source, Eric, Medline and PsycINFO) | TI ( adolescen* OR teen* OR young adults OR youth OR student ) OR AB (adolescen* OR teen* OR young adults OR youth OR student) NOT TI ( dropout* OR leaver OR patient ) NOT AB ( dropout* OR leaver* OR patient* ) TI ( education* OR school OR high?school OR secondary school OR post?primary OR school?based ) OR AB ( education* OR school OR high?school OR secondary school OR post?primary OR school?based ) TI ( prevention OR intervention OR program* ) AND AB ( prevention OR intervention OR program* ) TI suicid* OR AB suicid* AND TX ( suicid* ideation OR suicid* behavio* OR suicid* attempt* OR suicid* inten* OR suicid* thoughts OR self?injur* OR self?harm ) NOT TI ( review OR meta?analysis OR meta?regression OR sub?group analysis) |
| Web of Science | TOPIC: (adolescen* OR teen* OR young adults OR youth OR student) NOT TITLE: (dropout* OR leaver OR patient) AND TITLE: (education* OR school OR high?school OR secondary school OR post?primary OR school?based) AND TITLE: (prevention OR intervention OR program*) AND TITLE: (suicid* OR AB suicid*) AND ALL FIELDS: (suicid* ideation OR suicid* behavio* OR suicid* attempt* OR suicid* inten* OR suicid* thoughts OR self?injur* OR self?harm) NOT TITLE: (review OR meta?analysis OR meta?regression OR sub?group analysis) |
| Cochrane Central Register of Controlled Trials | (adolescen* OR teen* OR young adults OR youth OR student): TI,AB,KW AND (education* OR school OR high?school OR secondary school OR post?primary OR school?based): TI, AB ,KW AND (prevention OR intervention OR program*):TI, AB, KW AND (suicid* OR suicid*): TI, AB, KW in Trials |

Note: TI = Title. AB = Abstract. TX = All text. KW = Keyword.

| **Table S3** | | | | | | | |
| --- | --- | --- | --- | --- | --- | --- | --- |
| **Summary of PICOs, intervention and contextual factors for studies measuring suicidal ideation and/or attempts** | | | | | | | |
| **Study** | **Region & School details** | **Population & Sample Characteristics** | **Intervention/ Comparators** | **Intervention & stakeholder involvement** | **Outcome Measurement (follow-up) & OR (p-value)** | **Comparator Proportions** | **Risk of Bias** |
| Aseltine et al., 2007 | NA (Columbus, Georgia, western Massachusetts, and Hartford, Connecticut). Lower & middle class urban & suburban schools. | Multi-ethnic. Freshman, Sophomore, Junior & Senior. (^c^Female=2107 -51%). High school aged youth. | Universal/selective prevention (SOS): *n*=2039. Control (no intervention): *n*=2094. *n*^school^=9. | SOS targeted awareness, incorporated screening & taught signs of suicide and depression & ACT (2 days). External Interviewers. | YRBS (3 mnts). SA= (.63) (.05)^c^. SI=.84(.088)^b^ (calculated from SE & beta). | SA. SOS=60/1994, Control=92/2048 SI SOS= 201/ 1991,Control =235/2045^c.^Group sample sizes estimated (% baseline multiplied by follow-up). | Overall, BD1a, BD1b, BD2, BD3, BD5=Low. |
| Fekkes et al., 2016 | Europe (Netherlands),^.^ Public non-denominational schools^a^.^.^ | Grades 7-9, age range: 13-16 yrs (^c^male=832 – 60% of original sample n=1394). | Universal SEL intervention: *n*=283, *n*^school^=18.Waitlist control: *n*=229,*n*^school^=9. | Teacher-delivered Skills for Life targeted behavioural & health issues & social, moral & emotional skills (two school years in ln length). | Single-item questions (20 mnts) SA=.64(.92). SI=.90 (.70). | SA: SEL=11/283 Control=23/229 SI:SEL=54/283 control=60/229. | Overall. BD1a, BD1b, BD3=High risk BD2=Low. BD5=Some concerns. |
| Gould et al., 2005 | NA (New York State counties Nassau, Suffolk, and Westchester). Public coeducational & parochial boys’ schools. | 80.3% White. Grades 9-12. *M*=14.8, *SD*=1.2 yrs (58.1% of the original sample were male). | Selective prevention screening: *n*=731. Control (no screening): *n*=719. *n*^school^=6. | Screening (2 days) delivered by project child psychiatrist, psychologist or social workers. | Single item (postintervention). 1.2(.49). | Intervention= 34/731 - Control =28/719^c^ estimated by multiplying reported % by group baseline sample sizes. | Overall, BD1a, BD1b ,BD2,BD3,BD5=Low. |
| Perry et al., 2014 | Australia (Central West, New South Wales). Non-government, Catholic & independent schools. | Mostly White. School yrs 9 & 10, age range:13-16, *M*=14.75 yrs. Equal representation across genders. | Universal programme (HeadStrong): *n*=160, *n*^school^=4.Control (PDHPE): *n*=159, *n*^school^=2. | Headstrong is teacher-delivered & based on the PDHPE mental health and self-development curriculum (5–8 wks). | MFQ items measured SI (postintervention & 6 mnts). No adjustment effects. | Postintervention: Intervention =57/160.Control=52/159. 6 mnts:Intervention=41/136.Control=26/70.^b^ Dichotomised into “not true” & all other values. | Overall, BD3=High risk BD1a, BD1b, BD2, BD5=Low. |
| **Study** | **Region & School details** | **Population & Sample Characteristics** | **Intervention/ Comparators** | **Intervention & stakeholder involvement** | **Outcome Measurement (follow-up) & OR (p-value)** | **Comparator Proportions** | **Risk of Bias** |
| Perry et al., 2017 | Australia (Metropolitan Sydney). Selective & partially selective government schools. | Multi-ethnic gifted final year students *M*=16.7,*SD=*.51 yrs (female=63%) ) | Universal preventative depression (SPARX-R): *n*=242, *n*^school^=5. Control (LifeSTYLE): *n*=298, *n*^school^=5. | SPARX-R=gamified, CBT-based. LifeSTYLE=No mental health content (Both 5-7 wks) Teacher supervised intervention. | YRBS measured SA & SI.No adjusted effects. | 4 wks: Intervention=7/205 Control=8/196. 6 mnts: Intervention=3/126 Control =8/198.18 mnts: Intervention =1/39 Control=3/62. | Overall, BD1a=Some concerns BD1b,  BD2,  BD3, BD5=Low. |
| Poppelaars et al., 2016 | Europe (Netherlands). Unsegregated, low, intermediate & high secondary education schools. | Females with depressive symptoms, age range:11-16, *M*= 13.35,*SD*=.71 yrs. | OVK: *n*=50, *n*^school^=4. OVK/SPARX: *n*=56, *n*^school^=4. Active control: *n* =51, *n*^school^=4. | Indicated OVK & SPARX: depression prevention & CBT-based programmes. Psychologist supervised OVK (8 wks) SPARX: home based game. | Single CDI item measured SI (12 mnts). No adjusted effects. | 12 mnts OVK/SPARX = 3/56 OVK = 1/50 Control = 1/51^b^. | Overall, BD1a,BD1b,BD2,BD3,BD5=Low. |
| Schilling et al., 2014 | NA. High impact military middle schools. | White, Hispanic, Multi-ethnic (53% female). | SOS: *n*=299, *n*^school^=6. Wait-list control: *n*=87, *n*^school^=2. | School psychologists & counsellors & health teachers delivered SOS (2 days). | YRBS (3 mnts). No adjustment effects. | SI SOS=11/295 Control=9/86 SA SOS=5/296 Control =0/86^b^. Group follow-up were estimated using baseline% & follow-up. | Overall, BD1a =High risk BD1b, BD2, BD3, BD5 =Low. |
| Schilling et al., 2016 | NA (Connecticut). Technical &comprehensive schools. | White & Hispanic Ninth-grade students (58% male). | SOS: *n*=650, *n*^school^=9 Wait-list control: *n*=396 *n*^school^=8. | SOS. School counselling & social work staff presented the programme (2 days). | YRBS (3 mnts). SA =.36(.05)^c^. SI= .90 (.63)^b^ (calculated from SE & beta) | SI: SOS=45/650 Control: 36/396 SA: SOS=11/650 Control= 20/396. | Overall, BD1a=Some concerns BD1b,  BD2, BD3, BD5=Low |
| Shinde et al., 2020 | Asia (Bihar in India). Government secondary & higher, co-education schools size: >600, 301-600,101-300. | Grade 9 (55% male) age range=13-15, *M*=14.7,*SD* .9 yrs. | TSM: *n*=4786, *n*^school^ =24. SM: *n*=5084, *n*^school^=25. Control (Life skills programme AEP): *n*=5362, *n*^school^ =25 | Counsellor (SM)/teacher (TSM) delivered multicomponent universal health and school climate promotion intervention (SEHER). AEP (all arms) (104 wks). | SA Single-item (17 mnts) Adjusted: SM =1.11(.68) TSM=1.87 (.008). | No proportions reported. Crude OR and corresponding *p* values: TSM=1.96 (.004) SM=1.12 (.64). | Overall, BD1a, BD1b, BD2, BD3, BD5=Low |
| **Study** | **Region & School details** | **Population & Sample Characteristics** | **Intervention/ Comparators** | **Intervention & stakeholder involvement** | **Outcome Measurement (follow-up) & OR (p-value)** | **Comparator Proportions** | **Risk of Bias** |
| Vieland et al., 1991 | NA. Nonurban schools. Size: 750-1450. | Predominantly White students, age *M=*15.8 yrs (52% female). | Universal prevention intervention: *n*=174,  *n*^school^=2. Wait-list control: *n*=207, *n*^school^=2. | A teacher-delivered curriculum-based suicide prevention intervention (1.5 hrs) focusing on support, stress, peers & community resources. | Single item (18 mnts) No adjustment effects. | Intervention= 4/174 control =6/207.^b^ Proportions were estimated by multiplying rate of change: 2.5% (Intervention) & 2.7% (control) by groups. | Overall, BD1a=High risk BD1b, BD2, BD3, BD5=Low. |
| Wasserman et al., 2015 | Europe (Austria, Estonia, France, Germany, Hungary, Ireland, Italy, Romania, Slovenia, and Spain). Public schools. | Participants were from 10 European countries. Age range=14-16 *M=*14.8,*SD=*.8 yrs (59% female). | YAM universal prevention: *n*=2721, *n*^school^ =45. ProfScreen selective prevention: *n* =2764,*n*^schools^=43.QPR selective/indicated prevention:*n*=2692,*n*^school^=40.Wait-list control: *n*=2933 ,*n*^school^= 40. | YAM: Workshops targeting risk & protective factors. delivered by instructors. QPR: Gatekeeper training of teachers & school personnel. Profscreen: Screening & referral by health professionals (all 4 wks). | Paykel Hierarchical Suicidal Ladder measured SA &SI (3 &12 mnts). See Tables 2 & 3 in Wasserman et al., 2015. | See Tables 2 & 3 in Wasserman et al., 2015. | Overall, BD1a, BD1b, BD2, BD3, BD5=Low. |
| Wyman et al., 2010 | NA (Cobb County, Georgia, New York and North Dakota). Metropolitan & rural schools. | North American students (52%^c^ female). | Sources of Strength (universal/selective): *n*=1757, *n*^school^ =6. Wait-list control: *n* =918, *n*^school^ =6. | Peer leaders lead prevention messaging & encouraged youth to seek trusted adults (4 mnts). | Single-item (4mnts) Adjusted for baseline: SOS=77/ 1757.Control=47/918^b^(% of SI at follow-up by baseline). | SOS=232/1757 Control =124/918^b^ Proportions were estimated (% of SI at baseline and follow-up multiplied by baseline groups). | Overall, BD1a, BD1b, BD2, BD3, BD5=Low. |

Note: *n*=sample size. OR = odds ratio. β = beta. NI=No information. NA:North America *n*^school^=school sample size. SI=suicidal ideation. SA=suicide attempts. PICOs = Participants, Interventions, Control, Outcomes and Study Design. Hrs=hours. Wks=weeks. Mnts=Months. Yrs=Years. CDI = Children's Depression Inventory. CDC=Centers for Disease Control and Prevention. YRBS=Youth Risk Behaviour Survey. ACT= Acknowledge, care, and tell. MFQ=Moods and Feelings Questionnaire. PDHPE = Personal Development Health and Physical Education. CBT = cognitive behavioural therapy. OVK = Op Volle Kracht. YAM=Youth Aware of Mental Health Programme. QPR=Question, Persuade, Refer. SEL = Social and Emotional Learning. AEP = Adolescence Education Program. TSM=Teacher as SEHER Mitra. SM=SEHER Mitra. SEHER=Strengthening Evidence base on scHool-based intErventions for pRomoting adolescent health programme. BD=Bias domain. BD1= Bias domain 1: Bias arising from the randomization process. BD2= Bias domain2: Bias arising from the timing of identification and recruitment of individual participants in relation to timing of randomization. BD3=Bias domain 3: Bias due to deviations from intended interventions. BD4=Bias domain 4: Bias due to missing outcome data. BD5=Bias domain 5: Bias in selection of the reported result. ICC=Intracluster correlation coefficient. ^a^Corresponding author provided this information via email ^b^Estimated based on publication information and data sent by authors ^c^The value of the *p* value cut-off reported is used as exact *p* values were not reported.

**Table S4. Study characteristics adjustments**

| Study | Adjustments |
| --- | --- |
| Wasserman et al. 2015 | Age, sex, Strengths and Difficulties Questionnaire total score, not being born in the country of residence, parental job loss in the previous year, not living with both biological parents, and country of residence |
| Schilling et al. 2016 | Gender, ethnicity, grades, eligibility to receive free lunch, baseline reports of lifetime attempts and recent attempts |
| Aseltine et al. 2007 | Ethnicity, gender, grade, and study wave |
| Schilling et al. 2014 | Baseline suicidal ideation, lifetime attempt, gender, ethnicity, baseline parental military status and parental deployment, average grades, grade in school, post-test report of, professional treatment for depression or suicidality in the previous 3 months, English language learner status |
| Gould et al. 2005 | School |
| Fekkes et al. 2016 | Age, gender, education level and urbanisation |
| Shinde et al 2020 | Within-school clustering, stratification variables (school type, size, and nature), baseline cluster-level score of the outcome, and a priori fixed effects to account for age, gender, caste, marital status, and parents’ education and occupation |
| Wyman et al 2010 | Baseline |

**Table S5**

| **Meta-analysis and univariate meta-regressions for suicide attempts crude log ORs (k=13) (inclusion of Shinde et al 2020 teacher-led trial)** | | | | | | | |  |
| --- | --- | --- | --- | --- | --- | --- | --- | --- |
| **Meta-analysis** | **Log OR** | | **95% CI** | ***p* value** | | **Between I2 Within I2** | | |
| -.36 | | | -.81 to .09 | .11 | | 67.08 | 6.80 |  |
| **Meta-regressions** | | |  |  | |  |  |  |
| **Stakeholder**  **involvement (k[n])** | | | |  | |  |  |  |
| None (5[2]) | Reference group | | |  | | 63.65 | 10.29 | |
| Teacher or counsellor (4[3]) .25 -.70 to 1.21 | | | | | .60 |  |  |  |
| Multi-stakeholder (4[3]) -.13 -.68 to .43 | | | | | .66 |  |  |  |
| **Duration (k[n])** |  |  | | |  |  |  |  |
| 1 week ≤ (4[4]) | Reference group | | | |  | 75.76 | 4.27 |  |
| 4 weeks (6[1]) | .15 | -1.32 to 1.62 | | | .84 |  |  |  |
| 4 weeks>-104 weeks(3[2]) | .28 | -.96 to 1.52 | | | .66 |  |  |  |
| **Follow up (k[n])** |  |  | | |  |  |  |  |
| 3 months ≤ (6[4]) | Reference group | | | |  | 69.50 | 6.11 |  |
| 12 months (3[1]) | -.23 | -.75 to .29 | | | .39 |  |  |  |
| 17-20 months (4[3]) | .25 | -.73 to 1.23 | | | .62 |  |  |  |
| **Intervention primarily targets only STBs(k[n])** | | | | |  |  |  |  |
| No (3[2]) | Reference group | | | |  | 68.53 | 6.52 |  |
| Yes (10[5]) | -.28 | -1.27 to 0.71 | | | .58 |  |  |  |

Note: OR = odd ratio. k = number of effects. CI = confidence interval. Reference: Reference category.

**Table S6. Summary of univariate analyses of moderators for crude suicide attempt log odds ratio**

| **Effect moderator** | |  |  | **K(N)** | **OR** | **Contrast^1^ (95%CI)** | **QE** | **I2%between** | **I2%within** |
| --- | --- | --- | --- | --- | --- | --- | --- | --- | --- |
| **Stakeholder involvement** | | | |  |  |  | 14.83 | 63.57 | 6.04 |
| No school stakeholders | | | | 5(2) | .65 | Reference group |  |  |  |
| Teacher or counsellor | |  |  | 3(3) | .72 | .10 (-.66 to .87) |  |  |  |
| Multi-stakeholder | |  |  | 4(3) | .56^a^ | -.15 (-.64 to .34) | |  |  |
| **Intervention primarily targets only STBs** | | | | |  |  | 15.62 | 60.89 | .00 |
| No |  |  |  | 2(2) | .70 | Reference group |  |  |  |
| Yes |  |  |  | 10(5) | .63^a^ | -.11 (-.93 to .71) |  |  |  |
| **Follow up** |  |  |  |  |  |  | 15.36 | 63.44 | .00 |
| 3 months ≤ |  |  |  | 6(4) | .64 | Reference group |  |  |  |
| 12 months |  |  |  | 3(1) | .51^a^ | -.22 (-.68 to .23) |  |  |  |
| 17-20 months |  |  |  | 3(3) | .71 | .11 (-.72 to .94) |  |  |  |
| **Duration** |  |  |  |  |  |  | 14.94 | 69.89 | .00 |
| 1 week ≤ |  |  |  | 4(4) | .60 | Reference group |  |  |  |
| 4 weeks |  |  |  | 6(1) | .71 | .18 (-1.03 to 1.39) |  |  |  |
| 4 weeks> - 104 weeks |  |  |  | 2(2) | .68 | .14 (-.92 to 1.19) |  |  |  |

Note: K = number of effects. N = number of studies. OR = odds ratios. 1=contrasts are log odds ratios (LOR). QE = residual error. STBs = Suicidal thoughts and hebaviours. ^a^ = *p*<0.05. Contrast (LORs) are calculated from meta regressions with the intercept, using dummy variables. ORs are exponentiated LORs outputted from meta-regression models without the intercept

| **Effect moderator** | |  |  | **K(N)** | **OR** | **Contrast^1^ (95%CI)** | **QE** | **I2%between** | **I2%within** |
| --- | --- | --- | --- | --- | --- | --- | --- | --- | --- |
| **Stakeholder involvement** | | | |  |  |  | 7.03 | .00 | .00 |
| No school stakeholders | | | | 5(2) | .68^b^ | Reference group |  |  |  |
| Teacher or counsellor | |  |  | 3(3) | 1.06 | .45 (-.07 to .96) |  |  |  |
| Multi-stakeholder | |  |  | 4(3) | .63^a^ | -.08 (-.53 to .37) | |  |  |
| **Intervention primarily targets only STBs** | | | | | |  | 6.97 | .00 | .00 |
| No |  |  |  | 2(2) | 1.11 | Reference group |  |  |  |
| Yes |  |  |  | 10(5) | .67^b^ | -.51 (-1.04 to .02) |  |  |  |
| **Follow up** |  |  |  |  |  |  | 6.62 | 1.68 | .00 |
| 3 months ≤ |  |  |  | 6(4) | .70^a^ | Reference group |  |  |  |
| 12 months |  |  |  | 3(1) | .59^a^ | -.16 (-.59 to .26) |  |  |  |
| 17-20 months |  |  |  | 3(3) | 1.06 | .42 (-.11 to .95) |  |  |  |
| **Duration** |  |  |  |  |  |  | 6.60 | .00 | .00 |
| 1 week ≤ |  |  |  | 4(4) | .62^a^ | Reference group |  |  |  |
| 4 weeks |  |  |  | 6(1) | .70^a^ | .12 (-.27 to .52) |  |  |  |
| 4 weeks> - 104 weeks |  |  |  | 2(2) | 1.11 | .58 (-.00 to 1.16) |  |  |  |

**Table S7. Summary of univariate analyses of moderators for adjusted suicide attempt log odds ratio**

Note: K = number of effects. N = number of studies. OR = odds ratios. 1=contrasts are log odds ratios (LOR). QE = residual error. STBs = Suicidal thoughts and hebaviours. ^a^ = *p*< 0.01. ^b^ = *p*<0.001. Contrast (LORs) are calculated from meta regressions with the intercept, using dummy variables. ORs are exponentiated LORs outputted from meta-regression models without the intercept.

**Table S8**

| **Amendments to protocol** | **Rationale for amendments** |
| --- | --- |
| Inclusion of only cluster randomized control trial studies. | Only studies which employed a CRT design were included in this meta-analysis. The employment of CRT designs is necessary to establish intervention effectiveness when interventions are nested in contexts, which is the case when interventions are located in schools. It is unadvisable to synthesise studies with CRT designs with studies employing other study designs, as CRT designs contain additional sources of heterogeneity due to randomization unit and cluster size. When these additional sources of heterogeneity would likely interact with the intervention, it is particularly important to meta-analyse studies with CRT designs on their own (Donner & Klar, 2002). In the case of studies evaluating school-based interventions, where clustering represents the randomisation of schools and classes, there would likely be an interaction between the effectiveness of the intervention and clustering. |
| Exclusion of studies without potential to calculate crude ORs. | Studies which did not report/make available crude ORs unadjusted for study characteristics or data for potential calculation were excluded, as adjustment based on statistical relationships, overadjustment of effects and the pooling of effects with varying adjustments can incur bias and distorted inferences (Breslow, 1982;; Viechtbauer & Cheung, 2010). |
| Use of Cohen’s Kappa Coefficient instead of intraclass correlation co-efficient for absolute agreement. | Cohen’s Kappa Coefficient is a standard measure of agreement (Cohen, 1960) and was used as the outcomes were nominal. |
| Did not undertake the Grading of Recommendation Assessment, Development and Evaluation (GRADE) criteria. | The GRADE methodology was not undertaken for the meta-analysis as study design was homogeneous, risk of bias was measured by the Cochrane Collaboration Risk of Bias tool and consistency of SI and SA effects were measured using *I*2. |
| Use of R statistical language to conduct analyses. | R statistical programming was used instead of IBM SPSS statistics 26. |
| Omission of publication bias funnel plots. | Publication bias was not assessed visually by funnel plot symmetry as conducting these tests is not appropriate when conducting multi-level meta-analyses (Viechtbauer, 2010). |
| Did not assess risk of bias effects across study effect sizes. | Risk of bias assessment was not incorporated as a moderator and all studies were retained for meta-analysis as exclusion of studies based on bias may be a source of bias in itself (see Jüni, 2001). |
| Presentation of log ORs instead of risk ratios. | Dichotomous outcomes were expressed as log ORs as log ORs are a suitable effect outcome measure for dichotomous events which are rare (see Rucker et al., 2009), such as suicide outcomes. |
| Stakeholder involvement operationalisation. | Only involvement of stakeholders in intervention delivery was examined as data was not amenable to examine stakeholder involvement in design and assessment of the intervention. |

**References for Table S2**

Borenstein, M., Hedges, L. V., Higgins, J. P., & Rothstein, H. R. (2011). *Introduction to meta-analysis*. John Wiley and Sons.

Breslow, N. (1982). Design and analysis of case-control studies. *Annual review of public health, 3*(1), 29-54.

Cohen, J. (1960). A coefficient of agreement for nominal scales. *Educational and Psychological Measurement, 20*(1), 37-46. <https://doi.org/10.1177/001316446002000104>

Donner, A., & Klar, N. (2002). Issues in the meta-analysis of cluster randomized trials. *Statistics in Medicine, 21*(19), 2971-2980. <https://doi.org/10.1002/sim.1301>

Jüni, P. A., Douglas G , Egger, Matthias (2001). Assessing the quality of controlled clinical trials. *BMJ, 323*, 42-46. <https://doi.org/10.1136/bmj.323.7303.42>

Rucker, G., Schwarzer, G., Carpenter, J., & Olkin, I. (2009). Why add anything to nothing? The arcsine difference as a measure of treatment effect in meta-analysis with zero cells. *Statistics in Medicine, 28*(5), 721-738. <https://doi.org/10.1002/sim.3511>

Viechtbauer, W. (2010). Conducting meta-analyses in R with the metafor package. *Journal of Statistical Software*, 36(3), 1-48.

Viechtbauer, W., & Cheung, M. W. L. (2010). Outlier and influence diagnostics for meta-analysis. *Research Synthesis Methods, 1*(2), 112-125. <https://doi.org/10.1002/jrsm.11>
